# Chicken Egg White—Advancing from Food to Skin Health Therapy: Optimization of Hydrolysis Condition and Identification of Tyrosinase Inhibitor Peptides

**DOI:** 10.3390/foods9091312

**Published:** 2020-09-18

**Authors:** Pei-Gee Yap, Chee-Yuen Gan

**Affiliations:** Analytical Biochemistry Research Centre, Universiti Sains Malaysia, Penang 11800, Malaysia; peggy-yap@hotmail.com

**Keywords:** bioactive peptide, crossed D-optimal design, diphenolase inhibitory activity, egg white, monophenolase inhibitory activity, pigmentation, tyrosinase

## Abstract

Active fragments (bioactive peptides) from the chicken egg white proteins were expected to exert tyrosinase inhibitory activities in which skin hyperpigmentation could be prevented. Egg white was hydrolyzed by trypsin, chymotrypsin and the combination of both enzymes. The enzyme treatments achieved >50% degree of hydrolysis (DH) at substrate-to-enzyme (S/E) ratio of 10–30 (*w*/*w*) and hydrolysis time of 2–5 h. A crossed D-optimal experimental design was then used to determine the optimal enzyme composition, S/E ratio and hydrolysis time in order to yield hydrolysates with strong monophenolase and diphenolase inhibitory activities. The optimized conditions 55% trypsin, 45% chymotrypsin, S/E 10:1 *w*/*w* and 2 h achieved 45.9% monophenolase activity inhibition whereas 100% trypsin, S/E 22.13:1 *w*/*w* and 3.18 h achieved 48.1% diphenolase activity inhibition. LC/MS and MS/MS analyses identified the peptide sequences and the subsequent screening had identified 7 peptides (ILELPFASGDLLML, GYSLGNWVCAAK, YFGYTGALRCLV, HIATNAVLFFGR, FMMFESQNKDLLFK, SGALHCLK and YFGYTGALR) as the potential inhibitor peptides. These peptides were able to bind to H85, H94, H259, H263, and H296 (hotspots for active residues) as well as F92, M280 and F292 (stabilizing residues) of tyrosinase based on structure-activity relationship analysis. These findings demonstrated the potential of egg white-derived bioactive peptides as skin health therapy.

## 1. Introduction

The excess melanin production and deposition in the melanocytes and keratinocytes cause hyperpigmentation, leading to uneven skin tones. Tyrosinase (E.C. 1.14.18.1) plays a key role in melanin synthesis (melanogenesis) by catalyzing the rate-limiting hydroxylation of l-tyrosine to 3,4-dihydroxy-l-phenylalanine (l-DOPA) and the subsequent oxidation of l-DOPA to dopaquinone, via the monophenolase and diphenolase reactions, respectively [1]. The inhibition of tyrosinase using a nature-derived agent is hence of huge cosmeceutical demand, as some potent skin-lightening ingredients including hydroquinone and heavy metals have been ascribed with harmful side effects and banned for use in certain countries [2,3]. Since the discovery of a potential protein or peptide with tyrosinase inhibitory activity from lemon skin extract in 2006 [4], reports on peptide-based skin lightening agents has increased by leaps and bounds. The anti-pigmentation mechanisms of peptides include tyrosinase inhibition [5], copper chelation [6] and melanogenesis pathway regulation [7]. Peptide was also incorporated as an active ingredient in commercial skin lightening products such as β-White^TM^ (contains oligopeptide-68) and Melanostatine^TM^ 5 (contains nonapeptide-1) marketed by Lucas Meyer Cosmetics. The emergence of bioactive peptides as a new class of therapeutic agent is nonetheless endowed by their relatively small size, low toxicity, fast clearance and high specificity in inhibiting protein-ligand interactions [8].

Anti-pigmentation peptides have been discovered from various natural protein sources including rice bran [6,9] and marine microalgae [10]. Yet, there has been no relevant study on the anti-pigmentation effect of chicken egg white-derived peptides, although this readily available protein has been traditionally used as facial masks to boost skin health. It should be also noted that the active fragments are usually encrypted in the parent protein and required to be released in order to exhibit higher rate of the bioactivity. Enzymatic hydrolysis was therefore employed in this study to release the bioactive peptides from the egg white. In addition, the hydrolysis conditions such as the enzyme used, enzyme composition, substrate-to-enzyme (S/E) ratio and hydrolysis time in order to produce egg white hydrolysates that exhibit the highest monophenolase and diphenolase inhibitory activities should be optimized using D-optimal experimental design. The crossed D-optimal approach conjugates the mixture component (enzyme composition) and process factors (S/E ratio and hydrolysis time) in a single experimental design and generates a relatively smaller number of experimental runs, which has made it more viable in terms of cost and time, especially when each variable comes with multiple levels [11]. In fact, crossed D-optimal design had been successfully implemented to optimize different hydrolysis conditions for other purposes [12,13]. Therefore, this technique was used in this study.

Enzymes used in this study were trypsin and chymotrypsin due to their specific cleavage preferences, i.e., trypsin selectively cleaves at the C-terminal of arginine and lysine [14] whereas chymotrypsin predominantly cleaves at the C-terminal of the aromatic phenylalanine, leucine, methionine, tryptophan and tyrosine [15]. The peptides released may thus fulfil the characteristics of strong tyrosinase inhibitors such as the presence of an aromatic C-terminal residue tyrosine, tryptophan or phenylalanine [9,16] or one or more arginine in combination with phenylalanine, valine, alanine and leucine in the peptide sequence as delineated by Schurink, van Berkel, Wichers and Boeriu [17]. Therefore, the objectives of present study were to determine the optimum hydrolysis conditions of egg white using trypsin and/or chymotrypsin that yield the protein hydrolysates with tyrosinase inhibitory activities followed by peptide sequence identification as well as to investigate the structure-activity relationship between the identified inhibitor peptides and tyrosinase.

## 2. Materials and Methods

### 2.1. Chemicals

Egg white powder was purchased from a local market (Penang, Malaysia). Trypsin (EC 3.4.21.4, 10,000 U/mg) and α-chymotrypsin (EC 3.4.21.1, 40 U/mg) from bovine pancreas, tyrosinase (EC 1.14.18.1, 7164 U/mg) from *Agaricus bisporus* were purchased from Sigma-Aldrich. Other chemicals and reagents used were of analytical grade and purchased from Sigma-Aldrich unless otherwise stated.

### 2.2. Enzymatic Hydrolysis of Egg White Proteins

The enzymatic hydrolysis of egg white was conducted according to the protocol of Miguel, Recio, Gomez-Ruiz, Ramos and Lopez-Fandino [18] with modifications. Briefly, egg white powder was dissolved in 0.05 M sodium phosphate buffer (pH 7.8 or 8.0, depending on the digestive enzyme used) to 5 mg/mL and boiled at 95 °C for 30 min. Subsequently, designated enzyme treatments at various substrate-to-enzyme (S/E) ratios were added to the substrate solution and incubated for different durations at 37 °C with constant shaking of 300 rpm. The details of the experiment will be elaborated in Section 2.2.1 and Section 2.2.2. The resultant hydrolysate was then boiled at 95 °C for 30 min to terminate the reaction and centrifuged at 4500× *g* for 15 min. The supernatant was collected and stored at −20 °C until further analysis. Heat-inactivated enzymes were used as the control treatment.

#### 2.2.1. Single-Factor Experiment

The effects of enzyme composition, S/E ratio and hydrolysis time on the degree of hydrolysis (DH) and sodium dodecyl sulphate-polyacrylamide gel electrophoresis (SDS-PAGE) protein band profiling of egg white hydrolysate were studied as preliminary work to investigate the hydrolysis condition required to produce sufficient peptides from egg white. Below are the details of the experiment:

The effect of enzyme composition was investigated based on the hydrolysis of egg white using designated enzyme treatments: 100% trypsin (T), 100% chymotrypsin (C), and 50% trypsin + 50% chymotrypsin (T+C). Phosphate buffer at pH 7.8 was used for T whereas pH 8.0 was used for C and T+C treatments. All these compositions were used for the investigations of the effects of S/E ratio and hydrolysis time. During the investigation of the effect of S/E ratio, the ratio of 10:1, 20:1, 30:1, 40:1 and 50:1 (*w*/*w*) were studied whereas the hydrolysis time was fixed at 3 h. On the other hand, 0.5, 1, 2, 3, 4 and 5 h were studied during the investigation of the effect of hydrolysis period and the S/E ratio fixed at 30:1 (*w*/*w*).

The DH of the hydrolysate was determined by measuring the soluble protein content in 10% trichloroacetic acid (TCA) (Fisher Chemicals^TM^, Leicestershire, UK) according to the protocol of Baharuddin, Halim and Sarbon [19] with modifications. Briefly, the hydrolysate was dissolved in an equal volume of 20% TCA and incubated at room temperature for 30 min. The sample was then centrifuged at 3000× *g* for 10 min. The pellet was suspended in 0.5 mL 0.1 M NaOH and subjected to Bradford assay to determine the amount of soluble protein in the hydrolysate. Briefly, 5 µL of the hydrolysate was added with 250 µL of Bradford reagent and incubated at room temperature for 10 min. The absorbance was then measured at 595 nm using a spectrophotometer (Spectramax M5, Molecular Devices, San Jose, CA, USA). Each measurement was autozeroed against a blank containing Bradford reagent and 0.1 M NaOH. The DH was calculated using the following equation:

(1) $$\mathrm{Degree}\;\mathrm{of}\;\mathrm{hydrolysis}\;\left(\%\right)=\frac{A_{\mathrm{control}}-A_{\mathrm{sample}}}{A_{\mathrm{control}}}\times 100$$

where A_control_ denotes the absorbance of the system containing Bradford reagent, NaOH and undigested egg white powder; A_sample_ denotes the absorbance of the system containing Bradford reagent, NaOH and egg white hydrolysate.

To evaluate the protein profile after enzymatic hydrolysis, SDS-PAGE analysis was conducted using 4% stacking gel and 12% resolving gel. Briefly, 10 µL of sample was added with 10 µL 2× Laemmli buffer (Bio-Rad Laboratories Inc., Hercules, CA, USA) and 1 µL 2-mercaptolethanol followed by incubation at 95 °C for 15 min. Then, 10 µL of the mixture was loaded into the well and the set was run at 80 V for15 min followed by 120 V for 1 h. Opti-Protein XL Marker G266 (abm Inc., Richmond, BC, Canada) with a molecular weight range of 10 to 245 kDa was used as the standard protein marker. The gel was stained overnight using staining solution (50% ddH_2_O, 40% methanol, 10% acetic acid, 0.1% Coomassie Blue) and destained using destaining solution (50% ddH_2_O, 40% methanol, 10% acetic acid) until blue protein bands were visible against a clear background. The image of the gel was captured using Fujifilm LAS-3000 Imager (Fujifilm, Tokyo, Japan). The molecular weights of the protein bands were analyzed using Multi Gauge software version 3.0 (Fujifilm, Tokyo, Japan).

#### 2.2.2. Optimization of Hydrolysis Conditions for Monophenolase and Diphenolase Inhibitory Activities

Crossed D-optimal design was used to optimize the hydrolysis parameters to yield egg white hydrolysates with the highest tyrosinase inhibitory activities (i.e., the monophenolase inhibitory activity (Y) and diphenolase inhibitory activity (Z)). The enzyme composition (trypsin, X_1_; chymotrypsin, X_2_) represents the mixture component whereas S/E ratio (X_3_) and hydrolysis time (X_4_) represents the process factors. Based on the results of single-factor experiment (Section 2.2.1), the levels of the variables were chosen and coded, as shown in Table 1. These variables generated 28 experimental runs. Data analysis and calculation of predicted response were conducted using Design-Expert software (version 6.0, Minneapolis, MN, USA). Five confirmation experiments were performed to verify the optimized condition. 

### 2.3. Determination of Tyrosinase Inhibitory Activities

#### 2.3.1. Monophenolase Inhibitory Activity

The monophenolase inhibitory activity was performed according to Takahashi, Takara, Toyozato and Wada [20] with slight modifications. Briefly, 10 µL of sample and 180 µL of 50 mM potassium phosphate buffer (pH 6.8) containing 0.5 mM l-tyrosine were added to a 96-well plate and incubated at 30 °C for 10 min. The reaction was started by the addition of 1 µL tyrosinase (6250 U/mL) and immediately monitored at 470 nm at every 20 s for 15 min with a constant temperature of 30 °C throughout the reaction. Each measurement was autozeroed against a blank containing l-tyrosine. The monophenolase inhibitory activity is calculated as follows:

(2) $$\mathrm{Monophenolase}\;\mathrm{inhibitory}\;\mathrm{activity}\;\left(\%\right)=\frac{A_{\mathrm{control}}-A_{\mathrm{sample}}}{A_{\mathrm{control}}}\times 100$$

where A_control_ denotes the absorbance of the system containing tyrosinase and l-tyrosine; A_sample_ denotes the absorbance of the system containing tyrosinase, l-tyrosine and sample. 

#### 2.3.2. Diphenolase Inhibitory Activity

The diphenolase inhibitory activity was performed according to Takahashi, Takara, Toyozato and Wada [20] with slight modifications. Briefly, 10µL of sample and 180 µL of 50 mM potassium phosphate buffer (pH 6.8) containing 0.5 mM l-DOPA were added to a 96-well plate and incubated at 30 °C for 10 min. The reaction was started by the addition of 1 µL tyrosinase (6250 U/mL) and immediately monitored at 470 nm at every 10 s for 1 min with constant temperature 30 °C throughout the reaction. Each measurement was autozeroed against a blank containing l-DOPA. The diphenolase inhibitory activity is calculated as follows:

(3) $$\mathrm{Diphenolase}\;\mathrm{inhibitory}\;\mathrm{activity}\;\left(\%\right)=\frac{A_{\mathrm{control}}-A_{\mathrm{sample}}}{A_{\mathrm{control}}}\times 100$$

where A_control_ denotes the absorbance of the system containing tyrosinase and l-DOPA; A_sample_ denotes the absorbance of the system containing tyrosinase, l-DOPA and sample. 

### 2.4. Identification of Bioactive Peptides

The samples produced using the optimized parameters (Section 2.2.2) were subjected to LC/MS and MS/MS analyses using Easy-nLC II system (Thermo Scientific, San Jose, CA, USA) coupled with LTQ Orbitrap Velos. The chromatographic separation and mass spectrometry (MS) parameters were set up according to Siow and Gan [21]. Data acquisition was conducted using Xcalibur version 2.1. Peptide sequencing and identification based on the spectra acquired were performed using PEAKS Studio version 7.5 (Bioinformatics Solutions Inc., Waterloo, ON, Canada) [22]. The error mass tolerance allowed for precursor and fragmented ions were 0.1 and 0.8 Da, respectively. Enzyme was not specified in the peaks search against SwissProt2019 database and the false discovery rate (FDR) was estimated with decoy-fusion method. PeptideRanker web server (http://bioware.ucd.ie/, accessed on 6th July 2020) was used to screen for potential biologically active peptides where peptides with PeptideRanker score >0.5 were considered potentially active [23] and hence selected for further analysis. Protein-peptide docking was then performed using PepSite 2 web server (http://pepsite2.russelllab.org/, accessed on 7th July 2020) to predict the potential peptide binding sites on the protein molecule [24]. The server requires inputs for a protein structure in pdb format and a peptide sequence for prediction. The three-dimensional crystal structure of mushroom tyrosinase (PDB ID: 2Y9X) was obtained from the RCSB Protein Data Bank (PDB) at https://www.rcsb.org/ (accessed on 10th July). For peptide sequence input, peptides with >10 residues were split into equal portions since the maximum size of peptide accepted by PepSite 2 server was 10 residues. The predicted protein–peptide binding spots were ranked according to statistical significance where a *p*-value < 0.25 implies significant binding interaction.

### 2.5. Statistical Analysis

The study was performed in replicates. Statistical analysis was conducted using SPSS version 20.0 (SPSS Institute, Chicago, IL, USA). The results were analyzed using one-way ANOVA. *p*-value less than 0.05 implies a significant difference between sample means. T-test was conducted to analyze the significant (*p* < 0.05) difference between the experimental and predicted results for model validation.

## 3. Results and Discussion

### 3.1. Single-Factor Experiment

**Figure 1:**
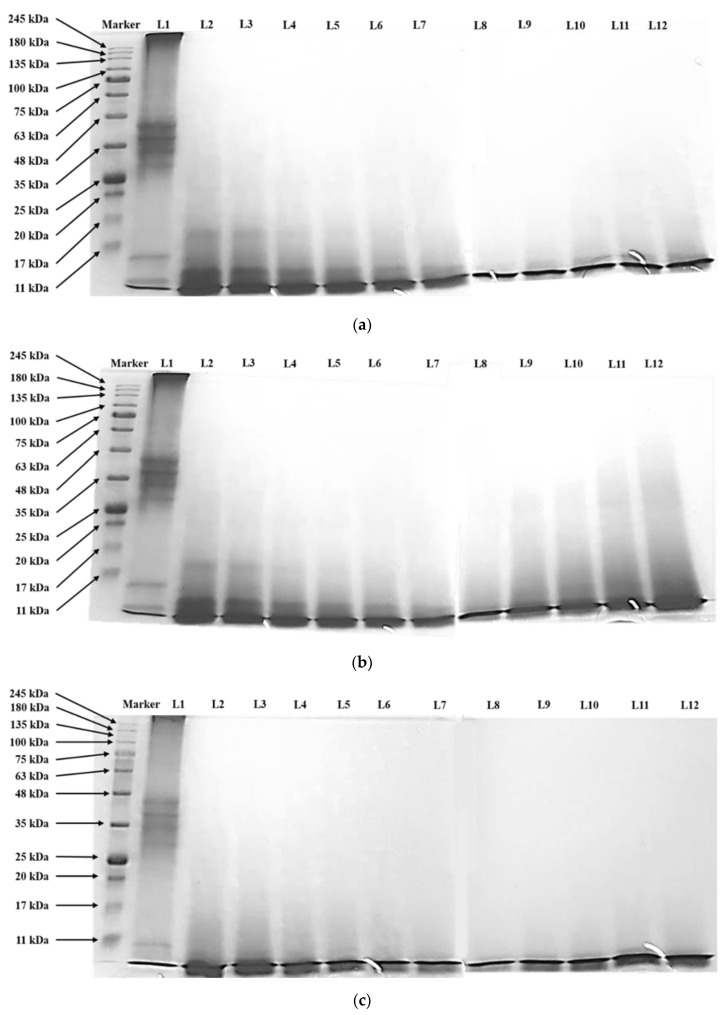
SDS-PAGE protein band profiling of albumin hydrolysate by (**a**) 100% trypsin, T; and (**b**) 100% chymotrypsin, C and (**c**) 50% trypsin + 50% chymotrypsin, T+C treatments at various substrate-to-enzyme (S/E) ratios and hydrolysis times. L1, control; L2, t = 0.5 h; L3, t = 1 h; L4, t = 2 h; L5, t = 3 h; L6, t = 4 h; L7, t = 5 h; L8, S/E = 10 (*w*/*w*); L9, S/E = 20 (*w*/*w*); L10, S/E = 30 (*w*/*w*); L11, S/E = 40 (*w*/*w*); L12, S/E = 50 (*w*/*w*).

The SDS-PAGE profile of the protein bands after enzymatic hydrolysis using different enzyme compositions, S/E ratios and hydrolysis times was shown in Figure 1. Generally, protein bands with MW ranging from 11–48 kDa and >245 kDa were observed in control treatments (L1). According to Abdou, Kim and Sato [25], the possible egg white proteins within or near the molecular weight range include ovomucin (230–8300 kDa), ovomacroglobulin (760–900 kDa), ovotransferrin (77.7 kDa), avidin (60 kDa), ovoinhibitor (49 kDa), ovoglobulin G3 (45 kDa), ovalbumin (44.5 kDa), ovoflavoprotein (32–36 kDa), ovoglobulin G2 (36 kDa), ovomucoid (28 kDa), ovoglycoprotein (24.4 kDa), lysozyme (14.4 kDa) and cystatin (12.7 kDa). Ovalbumin-related protein X and Y, on the other hand, share similar molecular weight of 50 kDa [26,27]. Notably, the >245 kDa bands were absent after enzymatic hydrolysis, suggesting the successful cleavage of large molecular weight proteins ovomucin and ovomacroglobulin into smaller protein fragments. For T treatment, the protein bands observed after hydrolysis were <17 kDa (Figure 1a). An 11–17 kDa band was observed at t = 0.5 and 1 h (Figure 1a, Lane 2 and 3) which is likely due to the presence of either lysozyme, cystatin or a subunit (15.6 kDa) of the tetrameric avidin. Protein bands of >25 kDa were also absent as the S/E ratio decreased from 50 to 10 (*w*/*w*) (Figure 1, Lane 8–12) where S/E 10 (*w*/*w*) treatment (Figure 1a, Lane 8) showed no observable bands at MW > 11 kDa. A <11 kDa band was found in all hydrolysates regardless of the hydrolysis time and S/E ratio, yet there were no reports on egg white proteins within this molecular weight range. This protein band may be contributed by a new, uncharacterized protein or the hydrolysis product from the aforementioned egg white proteins. Similar observations were recorded in C treatment (Figure 1b) yet 17–48 kDa protein bands were observed at S/E 40 and 50 (*w*/*w*) (Figure 1b, Lane 11 and 12), suggesting the incomplete hydrolysis of ovalbumin, ovoflavoprotein, ovomucoid or ovoglycoprotein when low amount of enzyme was used. Abeyrathne, Lee, Jo, Nam and Ahn [28] had reported the inability of 1% chymotrypsin to hydrolyze 20 mg/mL ovalbumin even up to 24 h. Apart from incomplete hydrolysis by chymotrypsin, the presence of ovoflavoprotein and ovomucoid could be attributed by their high thermal stability as boiling at 100 °C for 30 min could not denature the protein structures [25,29]. In contrast, T+C treatment showed complete digestion of large MW proteins (25–48 kDa) even when the shortest hydrolysis time at t = 0.5 h (Figure 1c, Lane 2) and the least amount of enzyme at S/E 50 (*w*/*w*) (Figure 1c, Lane 12) were used. This implies enzyme combination using trypsin and chymotrypsin was more effective in releasing smaller peptides from large protein molecules compered to individual enzyme treatments.

**Figure 2:**
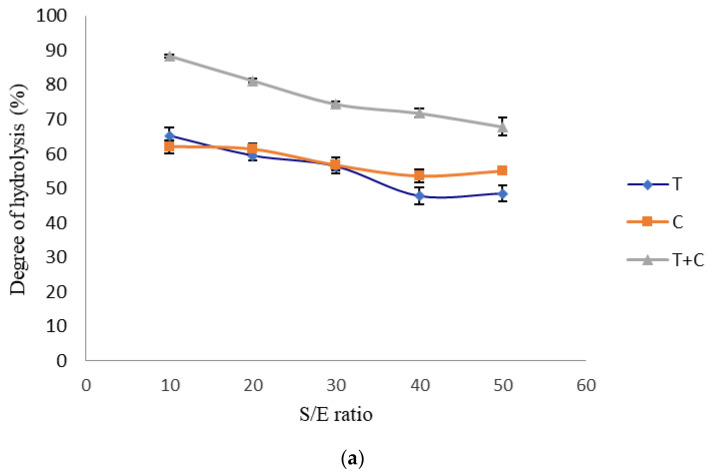

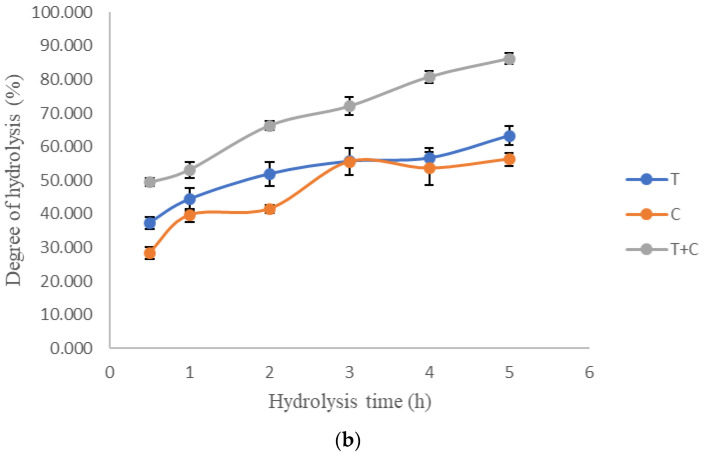
Effects of enzyme treatments (100% trypsin, T; 100% chymotrypsin, C and 50% trypsin + 50% chymotrypsin, T+C) on the degree of hydrolysis of egg white proteins at various (**a**) substrate-to-enzyme (S/E) ratio where the hydrolysis time is fixed at t = 3 h and (**b**) hydrolysis time where the S/E ratio is fixed at 30 (*w*/*w*). Results were reported as means with error bars representing the standard deviations of triplicate experiments.

DH is an indication of peptide bond cleavage in a protein hydrolysate and is vital in modulating the composition and properties of the peptides produced. The effects of different enzyme compositions, S/E ratio and hydrolysis time on the DH of egg white were summarized in Figure 2. Overall, it was observed that a lower S/E ratio and longer hydrolysis time contributed to higher DH in all T, C and T+C enzyme treatments. The highest DH was recorded in the T+C treatments at S/E ratio 10 (88.3 ± 0.4%; Figure 2a) and t = 5 h (86.1 ± 1.7%; Figure 2b). This could be due to the synergistic effect between the trypsin and chymotrypsin as the enzymes have different preferential cleavage sites. For example, the simultaneous treatment with trypsin and chymotrypsin had significantly reduced the time required for total hydrolysis of cheese whey proteins [30]. Chymotrypsin may cleave the bulky side chains of egg white proteins, exposing more cleavage sites for trypsin actions. Moreover, the resistance of albumin to trypsin digestion was overcome by boiling at 95 °C for 30 min before enzymatic hydrolysis. Heat treatment may have partially denatured or altered the tertiary structure of albumin, making the protein more accessible to both trypsin and chymotrypsin cleavage. A high DH corresponds to more peptide production and is often related to high bioactivity. For instance, Noh and Suh [31] who hydrolyzed egg white liquid using Alcalase, Neutrase, Protamex, Flavourzyme, Collupulin and Ficin had reported a positive correlation between DH and antioxidant activity. Alcalase hydrolysate produced at S/E 50 *w*/*w* and 24 h recorded the highest DH (43.2%) and free radical scavenging effects (82.5%) compared to other enzyme treatments. Moreover, high radical scavenging effect (ORAC value 1193.12 and DPPH value 19.05 Trolox EQ µmol g^−1^) were recorded when the DH of egg white were higher than 50% [32]. Similar conclusion was drawn by Chen, Chi, Zhao and Xu [33] where the antioxidant and angiotensin I-converting enzyme (ACE) inhibitory activities of egg white protein hydrolysate increased as the DH increased. Thus, together with the SDS-PAGE analysis result, the S/E ratio of 10–30 (*w*/*w*) and hydrolysis time of 2–5 h were selected for optimization study as these ranges recorded egg white hydrolysates with small peptides (MW < 17 kDa) and DH >50%.

### 3.2. Optimization of Tyrosinase Inhibitory Activities

The experimental and predicted responses of the 28 generated runs, presented as the mean of triplicate experiments, are shown in Table 2. There was a close agreement between the experimental and predicted responses. X_1_ was designated as the slack variable and removed from the model. This is because the summation of X_1_ and X_2_ equals 100%, hence a significant (*p* < 0.05) X_2_ reflects the significance of X_1_. Square root transformation of data was performed to normalize the data as suggested by the software.

Based on Table 2a, the experimental monophenolase inhibitory activity ranged from 15.2–48.0% under various test conditions, with the highest inhibition (47.989%) recorded at X_1_ = 75%, X_2_ = 25%, X_3_ = 20 *w*/*w* and X_4_ = 5 h. On the other hand, the experimental diphenolase inhibitory activity were ranging from 25.0–49.6% under various test conditions, with the highest inhibition (49.6%) recorded at X_1_ = 75%, X_2_ = 25%, X_3_ = 25 *w*/*w* and X_4_ = 2.75 h (Table 2b). Analysis of variance (ANOVA) was then conducted to evaluate the significance of the coefficient models at a 95% confidence interval (Table 3). The crossed reduced quadratic × cubic model was selected as it recorded a *p*-value of < 0.0001 for both monophenolase and diphenolase inhibitory activities. The insignificant lack of fit test *p*-value of 0.3348 (Table 3a) and 0.2906 (Table 3b) were observed for monophenolase and diphenolase inhibitory activities, respectively, indicating a well-fitted model that is adequate to describe the observed data. In addition, the values of coefficient of determination, R^2^ and adjusted R^2^ observed were 0.967 and 0.920, respectively (for monophenolase inhibitory activity), and 0.934 and 0.863, respectively (for diphenolase inhibitory activity), suggesting an excellent fit to the selected model. The coefficient of variation (CV) measures the dispersion of data around the mean. Both models had a low CV of 4.611 and 2.996%, respectively, implying a high precision and low degree of variation of the experiment performed.

The significant (*p* < 0.05) effect on the monophenolase inhibitory activity was contributed by the linear terms X_4_ and X_2_, quadratic term X_3_^2^ and various interaction terms X_2_X_3_, X_2_X_4_, X_3_X_4_, X_2_^2^X_3_, X_2_^2^X_4_, X_2_X_3_^2^ and X_3_^2^X_4,_ whereas, the significant (*p* < 0.05) effect on the diphenolase inhibitory activity was contributed by the linear terms X_4_ and X_2_, quadratic terms X_3_^2^ and X_4_^2^ alongside various interaction terms X_2_X_3_, X_2_X_4_, X_3_X_4_, X_2_^2^X_3_, X_2_^2^X_4_, X_2_X_3_^2^ and X_3_X_4_^2^. This suggested the hydrolysis time, enzyme compositions, as well as their interactions with S/E ratio, played a prominent role in the inhibition of these activities. To better fit the model, backward elimination step was carried out to eliminate the non-significant terms. The final response equations for monophenolase and diphenolase inhibitory activities in coded variables are given in Equations (4) and (5), respectively.

(4) $$\begin{array}{cc} \sqrt{Monophenolase\;inhibitory\;activity} & =6.269-2.711x_{2}+1.395x_{4}-1.084x_{3}^{2}+0.612x_{4}^{2}-3.157x_{2}x_{3}\\ & -2.128x_{2}x_{4}+0.289x_{3}x_{4}+2.835x_{2}^{2}x_{3}+2.334x_{2}^{2}x_{4}+3.45x_{2}x_{3}^{2}\\ & -0.926x_{2}x_{4}^{2}-0.938x_{3}^{2}x_{4} \end{array}$$

(5) $$\begin{array}{cc} \sqrt{Diphenolase\;inhibitory\;activity} & =6.943-1.258x_{2}-0.269x_{4}-0.952x_{3}^{2}-0.269x_{4}^{2}+1.301x_{2}x_{3}\\ & -1.472x_{2}x_{4}-0.139x_{3}x_{4}-1.267x_{2}^{2}x_{3}+2.244x_{2}^{2}x_{4}+1.726x_{2}x_{3}^{2}\\ & -0.226x_{3}x_{4}^{2} \end{array}$$

### 3.3. Verification of Predictive Models

Various combination of hydrolysis parameters was suggested to verify the suitability of the predictive models. Taking into consideration the cost, efficiency and feasibility of the experiment, the optimal condition (desirability value of 0.949, which indicating that the suggested condition is close to the desired process condition-minimum amount of enzymes, minimum hydrolysis time and maximum inhibitory activities) to achieve monophenolase inhibitory activity of 45.9% corresponded to 55% trypsin, 45% chymotrypsin, S/E ratio 10:1 (*w*/*w*) and hydrolysis time 2 h was examined. The experimental value of 45.3% was found close with no significant (*p* > 0.05) difference with the predicted value (45.9%). The DH determined was 84.8 ± 1.8%. For diphenolase inhibitory activity, the optimal condition suggested was 100% trypsin, 0% chymotrypsin, S/E ratio 22.13:1 (*w*/*w*) and hydrolysis time 3.18 h. The experimental value of 48.1% was found close with no significant (*p* > 0.05) difference with the predicted value (48.1%). The degree of hydrolysis determined was 64.0 ± 2.1%. Therefore, this model was valid for the optimization of monophenolase and diphenolase inhibitory activities from egg white. It was also observed that in monophenolase inhibitory activity optimization, even though 48.0% was achieved by sample run 23 (Table 2), 5 h of hydrolysis time was required, whereas 2 h of hydrolysis time could achieve 45.3% (the difference by only 2.7%) in the optimized condition. In the optimization of diphenolase inhibitory activity, the optimized sample achieved 48.1% activity by using only trypsin (lower in cost) compared to the sample run 17 (49.6%) in which the difference was only 1.5%. Therefore, it was suggested that the optimization process had managed to achieve the goal of study.

### 3.4. Identification of Bioactive Peptides

There were 139 and 189 peptides identified for monophenolase and diphenolase inhibitory activities, respectively, using PEAKS Studio (Appendix A Table A1). For monophenolase inhibitory activity, 76, 36, 4, 7, 11, 4 and 1 peptides were identified from ovalbumin, ovotransferrin, ovomucoid, ovalbumin-related protein X, ovalbumin-related protein Y, Ovomucin, and cystatin, respectively, where they were found to achieve 77%, 45%, 42%, 38%, 32%, 16% and 12% coverages of the corresponding protein sequences (Appendix A Table A1a). For diphenolase activity, 81, 54, 9, 12, 4, 8, 20 and 2 peptides identified were found to match 81%, 63%, 54%, 38%, 29%, 28%, 26% and 2% sequence coverages of ovalbumin, ovotransferrin, lysozyme, ovomucoid, ovalbumin-related protein X, ovalbumin-related protein Y, Ovomucin and ovostatin, respectively (Appendix A Table A1b). Subsequently, PeptideRanker web server was used to screen for potential biologically active peptides since the likeliness of being bioactive is usually governed by specific structural characteristics of peptide [23]. The use of PeptideRanker for initial screening and prediction had been proven successful to identify bioactive peptides with wide array of bioactivities [34,35,36]. Therefore, there were 7 and 21 peptides (PeptideRanker score >0.5) shortlisted for monophenolase and diphenolase inhibitory activities, respectively. The shortlisted peptides were further subjected to structure-activity relationship analysis with mushroom tyrosinase using the PepSite2 web server.

The *p*-values of mushroom tyrosinase-peptide binding interactions predicted by PepSite 2 web server were summarized in Table 4. A smaller *p*-value signifies higher potential of peptide binding to the enzyme. The smallest *p*-value was recorded by ADHPF (0.002658), AFKDEDTKAMPF (0.02053) and ILELPFASGDLLML (0.03464) whereas the largest *p*-value was recorded by DGSGGCIPK (0.1274) for monophenolase inhibitory activity (Table 4a). For diphenolase activity, SDFHLFGPPGK (0.009412), FDGRSR (0.01312) and FNCSSAGPGAIGSEC (0.01614) were among the peptides with the smallest *p*-values whereas YFGYTGALR had recorded the largest *p*-value of 0.2364 (Table 4b). Overall, all peptides showed significant (*p* < 0.25) binding interactions with mushroom tyrosinase. Notably, phenylalanine, leucine and alanine were frequently observed in the peptide sequences. Phenylalanine may act as the pseudo-substrate of tyrosinase since it is structurally identical to tyrosine, the natural substrate of tyrosinase. Besides, the hydrophobic side chains of leucine and alanine may interact directly with the hydrophobic binding pocket of tyrosinase to cause enzyme inhibition. According to Strothkamp, Jolley and Mason [37], mushroom tyrosinase is a tetramer comprising of two H subunits and two L subunits. The L subunit is the product of *Orf239342* gene and possesses a lectin-like structure, hence annotated as mushroom tyrosinase associated lectin-like protein. It comprises residues 9–28 and 35–150 of ORF239342 protein and is arranged into 12 antiparallel β-strands that is located away from the tyrosinase catalytic site, suggesting an insignificant role in enzyme activity [38]. The L subunit was postulated to provide innate immunity against bacterial infection [39] and act as a cofactor in melanin production [40]. In contrast, the H subunit originates from *ppo3* gene and covers residues 2–392 of PPO3. This subunit is made up of 13 α-helices, 8 short β-strands and loops that structured the catalytically essential tyrosinase core domain [38,41]. The H subunit houses a binuclear copper active site where copper A is coordinately bonded with H61, H85 and H94 whereas copper B to H 259, H263 and H296. The 6 histidine residues form a hydrophobic binding pocket at the bottom of the H subunit and H263 is postulated to regulate proper orientation of incoming substrate [38]. The structural rigidity of the binding pocket is maintained by several interactions between the 6 histidine and their neighboring residues. For instance, the side chain rotational freedom of H85 is restricted through the formation of a thioether bond with the side chain of C83. This thioether bond stabilizes H85 and is also suggested to optimize redox potential as well as to facilitate rapid electron transfer for the redox reactions occurring in the binuclear copper site [38,42]. Furthermore, the presence of F90 confers structural constraints to H94, H259 and H296 while F292 limits the side chain flexibility of H61, H263 and H296 [38]. The interaction between M280 and the aromatic ring of histidines also stabilizes the protein structure [43] and this residue may aid in copper incorporation into the binding pocket [44]. Notably, the ILELPFASGDLLML for monophenolase inhibitory activity (Table 4a) and GYSLGNWVCAAK, YFGYTGALRCLV, HIATNAVLFFGR, FMMFESQNKDLLFK, SGALHCLK and YFGYTGALR for diphenolase inhibitory activity (Table 4b) were found to interact with H61, H85, H94, H259, H263, and H296 (hotspot residues) and F92, F292 and M280 (stabilizing residues) of mushroom tyrosinase. The peptide interactions with the hotspot residues may weaken or hinder enzyme binding with its putative substrate whereas peptide interactions with the stabilizing residues may disrupt the integrity of active site, which reduces the catalytic potency of the enzyme. Thus, ILELPFASGDLLML, GYSLGNWVCAAK, YFGYTGALRCLV, HIATNAVLFFGR, FMMFESQNKDLLFK, SGALHCLK and YFGYTGALR represent potential tyrosinase inhibitory peptides.

On the other hand, majority of the peptides were also found to bind to Y140, W386 and H390 (Table 4) which were not within the mushroom tyrosinase substrate binding pocket. Hassani Hagnbeen and Fazli [45] reported two mixed-type inhibitors of tyrosinase, phthalic acid and cinnamic acid, each bound to different binding sites of the enzyme. For instance, phthalic acid formed hydrogen bonds with W136, W141 and G149 and van der Waals interactions with D137, W138, G139, Y140, F147 and F224 whereas cinnamic acid form hydrogen bonds with Q307 and D312 and van der Waals interactions with T308, Y311, V313, Y314, E356 and W358. Jung et al. [46] also reported a mixed-type tyrosinase inhibitor, (E)-2-(2,4-dihydroxybenzylidene)-2,3-dihydro-1H-inden-1-one (BID3) which formed a hydrogen bond with Y140 and interacted hydrophobically with L24, F147 and I148. These findings suggest potential peptide interaction with non-specific binding site of the enzyme since the allosteric site of mushroom tyrosinase has yet to be identified.

## 4. Conclusions

In this study, egg white has been proven to be more than just a food component. The optimization of enzymatic hydrolysis conditions, LC/MS MS/MS peptide identification and sequencing followed by structure-activity relationship analyses had corroborated the potential of this food protein as a source for the production of anti-tyrosinase peptides to prevent skin hyperpigmentation. Nonetheless, the monophenolase and diphenolase inhibitory peptides identified will next be chemically synthesized and validated for their in vitro anti-tyrosinase efficacies before proceeding to in vivo assays to examine their effects on the melanogenesis pathway regulatory proteins.

## Figures and Tables

**Table 1:** Parameters and levels for crossed D-optimal design of the monophenolase and diphenolase inhibitory activities.

Variable	Coded Variable	Coded Variable Level				
		−1	−0.5	0	0.5	1
Trypsin composition (%)	X_1_	0	25	50	75	100
Chymotrypsin composition (%)	X_2_	0	25	50	75	100
S/E ratio (*w*/*w*)	X_3_	10	15	20	25	30
Hydrolysis time (h)	X_4_	2	2.75	3.5	4.25	5

**Table 2:** Crossed D-optimal experimental design with the actual and predicted responses for (**a**) monophenolase and (**b**) diphenolase inhibitory activities.

			S/E Ratio, X_3_	Hydrolysis Time, X_4_ (h)	(a)		(b)	
Run	Enzyme Composition (%)				Monophenolase Inhibitory Activity (%)		Diphenolase Inhibitory Activity (%)	
	Trypsin, X_1_	Chymotrypsin, X_2_			Experimental (y_1_)	Predicted * (y_0_)	Experimental (z_1_)	Predicted ^#^ (z_0_)
1	100	0	10	5	32.5 ± 2.8	35.6	30.7 ± 1.7	33.6
2	50	50	30	2	20.8 ± 1.8	20.2	44.3 ± 4.5	44.1
3	0	100	20	3.5	15.2 ± 1.4	12.7	31.4 ± 3.5	32.1
4	0	100	10	2	31.6 ± 2.4	30.5	34.2 ± 4.0	33.8
5	100	0	30	2	24.7 ± 1.3	24.3	35.7 ± 2.1	36.1
6	0	100	30	5	39.1 ± 3.4	38.5	40.6 ± 1.8	40.9
7	0	100	30	2	18.9 ± 1.8	19.6	30.2 ± 2.3	31.1
8	100	0	20	2	29.5 ± 4.7	30.7	45.5 ± 3.6	48.1
9	0	100	30	2	20.0 ± 3.8	19.6	32.0 ± 0.5	31.1
10	50	50	10	5	37.2 ± 2.5	37.5	32.8 ± 0.9	31.7
11	0	100	10	2	30.2 ± 5.0	30.5	33.7 ± 1.7	33.8
12	50	50	30	5	26.1 ± 3.7	25.3	28.8 ± 2.7	30.0
13	100	0	30	3.5	26.3 ± 3.5	27.3	38.1 ± 2.4	37.3
14	100	0	10	2	30.3 ± 4.5	32.8	35.8 ± 3.9	35.6
15	50	50	20	2	15.8 ± 3.8	16.9	44.7 ± 3.9	42.5
16	50	50	30	3.5	19.2 ± 2.8	21.0	43.4 ± 3.8	44.3
17	75	25	25	2.75	26.1 ± 3.5	23.5	49.6 ± 3.2	47.0
18	0	100	20	5	21.8 ± 3.6	23.9	36.2 ± 3.4	35.1
19	25	75	15	2.75	19.0 ± 2.1	20.9	31.8 ± 2.5	35.0
20	0	100	30	5	38.4 ± 0.6	38.5	41.6 ± 2.2	40.9
21	0	100	10	5	43.4 ± 4.0	41.5	46.6 ± 2.0	48.7
22	0	100	10	3.5	37.4 ± 4.5	39.7	41.7 ± 2.7	39.3
23	75	25	20	5	48.0 ± 4.1	48.1	35.9 ± 2.8	34.5
24	100	0	30	5	44.7 ± 0.2	44.4	25.0 ± 2.3	25.2
25	100	0	10	5	38.7 ± 0.7	35.6	35.7 ± 4.2	33.6
26	100	0	10	2	35.2 ± 1.3	32.8	36.9 ± 1.4	35.6
27	50	50	10	2	47.2 ± 2.2	45.9	37.7 ± 2.5	38.6
28	75	25	15	3.5	36.1 ± 1.4	34.6	39.1 ± 1.7	39.7

Note: Data is presented as the mean ± standard deviation of triplicate experiments; * predicted using Equation (4); ^#^ predicted using Equation (5).

**Table 3:** ANOVA for crossed reduced quadratic × cubic model: estimated regression model of the relationship between the mixture component (X_1_, X_2_), process variables (X_3_, X_4_) and the response variables (**a**) monophenolase inhibitory activity (Y) and (**b**) diphenolase inhibitory activity (Z).

Source	Sum of Squares	DF	Mean Square	F Value	Prob > F
**(a) Monophenolase inhibitory activity (Y)**					
Model	20.668	16	1.292	20.447	<0.0001
X_2_	1.678	1	1.678	26.563	0.0003
X_3_	0.009	1	0.009	0.139	0.7167
X_4_	4.155	1	4.155	65.766	<0.0001
X_2_^2^	0.075	1	0.075	1.189	0.2988
X_3_^2^	1.469	1	1.469	23.260	0.0005
X_4_^2^	0.258	1	0.258	4.079	0.0685
X_2_X_3_	1.728	1	1.728	27.346	0.0003
X_2_X_4_	0.973	1	0.973	15.396	0.0024
X_3_X_4_	0.873	1	0.873	13.812	0.0034
X_2_^2^X_3_	1.489	1	1.489	23.572	0.0005
X_2_^2^X_4_	1.222	1	1.222	19.342	0.0011
X_2_X_3_^2^	5.107	1	5.107	80.846	<0.0001
X_2_X_4_^2^	0.217	1	0.217	3.442	0.0905
X_3_^2^X_4_	2.024	1	2.024	32.038	0.0001
X_3_X_4_^2^	0.005	1	0.005	0.071	0.7943
X_2_X_3_X_4_	0.073	1	0.073	1.148	0.3069
Residual	0.695	11	0.063		
Lack of Fit	0.447	6	0.075	1.507	0.3348
Pure Error	0.247	5	0.049		
Cor Total	21.362	27			
R^2^	0.967				
Adjusted R^2^	0.920				
C.V.	4.611				
**(b) Diphenolase inhibitory activity (Z)**					
Model	6.078	14	0.434	13.106	<0.0001
X_2_	0.185	1	0.185	5.573	0.0345
X_3_	0.071	1	0.071	2.143	0.1669
X_4_	0.519	1	0.519	15.658	0.0016
X_2_^2^	0.010	1	0.010	0.316	0.5833
X_3_^2^	1.283	1	1.283	38.730	<0.0001
X_4_^2^	0.214	1	0.214	6.454	0.0246
X_2_X_3_	0.235	1	0.235	7.104	0.0194
X_2_X_4_	0.385	1	0.385	11.632	0.0046
X_3_X_4_	0.258	1	0.258	7.799	0.0152
X_2_^2^X_3_	0.263	1	0.263	7.939	0.0145
X_2_^2^X_4_	1.060	1	1.060	31.990	<0.0001
X_2_X_3_^2^	1.771	1	1.771	53.473	<0.0001
X_3_X_4_^2^	0.314	1	0.314	9.481	0.0088
X_2_X_3_X_4_	0.040	1	0.040	1.198	0.2936
Residual	0.431	13	0.033		
Lack of Fit	0.315	8	0.039	1.696	0.2906
Pure Error	0.116	5	0.023		
Cor Total	6.508	27			
R^2^	0.934				
Adjusted R^2^	0.863				
C.V.	2.996				

**Table 4:** List of potential biologically active egg white-derived peptides shortlisted for (**a**) monophenolase and (**b**) diphenolase inhibitory activities using PeptideRanker web server and their potential binding sites on mushroom tyrosinase predicted using PepSite 2 web server.

No.	Peptide Sequence	Egg Protein Fragment	Peptide Length	Potential Binding Sites of Mushroom Tyrosinase (PDB ID: 2Y9X)	PepSite 2 *p*-Value
**(a) Monophenolase inhibitory activity**					
1	ADHPF	Ovalbumin	5	Y140, K389, H390	0.002658
2	AFKDEDTKAMPF	Ovalbumin	12	N22, F135, D137, Y140. R301, P366, D367, W386, H390, Y391	0.02053
3	ILELPFASGDLLML	Ovalbumin-related protein X	14	Y36, L40, F54, G58, **H61**, **H85**, F90, **H94**, W101, Q133, **H259**, **H263**, *M280*, H285, A286, A287, F288, D289, P290, *F292*, W293, **H296**	0.03464
4	DKLPGFGD	Ovalbumin	8	Y140, P370, Y382, W386, K389, H390	0.05277
5	FDKLPGFGD	Ovalbumin	9	Y140, P370, Y382, W386, K389, H390	0.0722
6	FDKLPGFGDSIEAQCGTSVN	Ovalbumin	20	Y140, T233, R301, M309, D367, P370, Y382, N384, W386, H388, K389, H390	0.08107
7	DGSGGCIPK	Ovomucin	9	N22, F135, D137, Y140, R301, D367, Y382, W386, K389, H390	0.1274
**(b) Diphenolase inhibitory activity**					
1	SDFHLFGPPGK	Ovotransferrin	11	Y140, R301, D367, Y382, W386, H390	0.009412
2	FDGRSR	Ovomucin	6	D137, R301, P366, D367, W386, H390, Y391	0.01312
3	FNCSSAGPGAIGSEC	Ovomucin	15	N22, F135, D137, Y140, R301, P366, D367, W386, H390, Y391	0.01614
4	MYQIGLFR	Ovalbumin	8	D137, Y140, R301, D367, P370, Y382, W386, H390, Y391	0.01832
5	GYSLGNWVCAAK	Lysozyme	12	**H61**, N81, Y82, C83, T84, **H85**, F90, W93, **H94**, R95, Y97, E98, E256, **H259**, **H263**, **M280**, V283, A286, A287, **F292**, W293, **H296**	0.01891
6	DLLFKDSAIMLK	Ovotransferrin	12	D137, Y140, R301, D367, Y382, W386, K389, H390, Y391	0.02538
7	CQLCQGSGGIPPEK	Ovotransferrin	14	D137, Y140, R301, P366, D367, Y382, W386, H390, Y391	0.02578
8	ADHPFLF	Ovalbumin	7	Y140, R301, P366, D367, F368, P370, W386, H390	0.03051
9	SGAFHCLK	Ovotransferrin	8	Y140, Y382, W386, H390	0.03994
10	YFGYTGALRCLV	Ovotransferrin	12	**H61**, **H85**, **H94**, Y97, Y140, **H259**, **H263**, *M280*, V283, A287, *F292*, W293, H295, **H296**, V299, R301, D367, Y382, W386, H390	0.04488
11	HIATNAVLFFGR	Ovalbumin	12	G58, **H61**, C83, **H85**, F90, Typ93, **H94**, Y97, D137, Y140, **H259**, **H263**, *M280*, H285, A286, A287, F288, D289, *F292*, W293, **H296**, R301, D367, W386, H390, Y391	0.04707
12	FKDEDTQAMPFR	Ovalbumin	12	D137, Y140, R301, P366, D367, W386, H390, Y391	0.05594
13	FMMFESQNKDLLFK	Ovotransferrin	14	**H61**, N81, Y82, C83, T84, **H85**, F90, W93, **H94**, Y97, D137, Y140, **H259**, **H263**, *M280*, A286, A287, *F292*, W293, **H296**, R301, P366, D367, W386, H390, Y391	0.06287
14	FDKLPGFGD	Ovalbumin	9	Y140, P370, Y382, W386, K389, H390	0.0722
15	SMLVLLPDEVSGLEQLESIINFEK	Ovalbumin	24	D137, Y140, R301, D367, P370, Y382, W386, K389, H390, Y391	0.08195
16	SGYSGAFHCLK	Ovotransferrin	11	Y140, R301, P366, D367, Y382, W386, H390	0.0849
17	SGGQFSLTSTVKVC	Ovomucin	14	D137, Y140, R301, D367, W386, H390, Y391	0.08667
18	SGALHCLK	Ovotransferrin	8	**H61**, N81, Y82, C83, T84, **H85**, W93, **H94**, R95, Y97, E98, **H259**, **H263**, A286, A287, *F292*, W293, **H296**	0.1247
19	SSCICS	Ovomucin	6	N22, F135, D137, R301, P366, D367, W386, H390	0.1938
20	SSCEDCVCT	Ovomucin	9	D137, Y140, R301, D367, W386, H390, Y391	0.2086
21	YFGYTGALR	Ovotransferrin	9	**H61**, **H85**, **H94**, Y97, **H259**, **H263**, *M280*, V283, A286, Als287, *F292*, W293, H295, **H296**, V299	0.2364

Note: Underlined residues are actively involved in binding interaction with mushroom tyrosinase; Residues in bold indicate mushroom tyrosinase hotspots; Residues in italics indicate stabilizing residues.

## Appendix A

**Table A1:** List of egg white-derived peptides for (**a**) monophenolase and (**b**) diphenolase inhibitory activities with their corresponding PeptideRanker scores.

No.	Egg Protein Sequence Coverage	Peptide	Peptide Sequence Number	Score
**(a) Monophenolase inhibitory activity**				
**1**	Ovalbumin (77%)	ADHPF *	361–365	0.8592
**2**		FDKLPGFGD *	60–68	0.6817
**3**		FDKLPGFGDSIEAQCGTSVN *	60–79	0.5429
**4**		AFKDEDTKAMPF *	188–199	0.5332
**5**		DKLPGFGD *	61–68	0.5174
**6**		MSALAM	36–41	0.4941
**7**		RGGLEPINF	127–135	0.4842
**8**		YPILPEYL	112–119	0.4732
**9**		AFKDEDTQAMPF	188–199	0.4636
**10**		VLLPDEVSGLEKLESIINF	244–262	0.4494
**11**		QVLLPDEVSGLEQLESIINF	243–262	0.4335
**12**		KDEDTQAMPF	190–199	0.4113
**13**		FDKLPGFGDSIEAQ	60–73	0.4067
**14**		YPILPEYLQCVKELY	112–126	0.3960
**15**		VLLPHEVSGLEQLESIINF	244–262	0.3712
**16**		LVQLPDEVSGLEQLESIINF	243–262	0.3593
**17**		LPDEVSGLEQLESIINF	246–262	0.3544
**18**		MAMGITDVF	299–307	0.3250
**19**		SSSANLSGISSAESLK	308–323	0.3207
**20**		LVLLPDEVSGLEQLESIINF	243–262	0.3189
**21**		VHHANENIFY	21–30	0.3132
**22**		VTEQESKPVQMMYQIGLF	210–218	0.3126
**23**		VHHANENIF	21–29	0.2953
**24**		GGLEPINFQTAADQAR	128–143	0.2810
**25**		VLLPDEVSGLEQLESIINFE	244–263	0.2738
**26**		VASMASEKMK	220–229	0.2728
**27**		AAHAEINEAGR	330–340	0.2679
**28**		AEERYPILPEYL	108–119	0.2648
**29**		YPILPEYLQ	112–120	0.2638
**30**		ISQAVHAAHAEINEAGR	324–340	0.2599
**31**		SANLSGISSAESLK	310–323	0.2537
**32**		SWVESQTNGIIR	148–159	0.2523
**33**		AFKDEDTQAMP	188–198	0.2456
**34**		SGISSAESLK	314–323	0.2242
**35**		MAMGITDVFSSSANLSGIS	299–317	0.2239
**36**		INSWVESQTNGIIR	146–159	0.2193
**37**		LYAEERYPILPEYL	106–119	0.2015
**38**		SGISSAESL	314–322	0.1982
**39**		VLLPDEVSGLEQLESIINFEK	244–264	0.1885
**40**		VLQPSSVHSQTAM	161–173	0.1836
**41**		VLLPDEVSGLEQL	244–256	0.1835
**42**		LSGISSAESLK	313–323	0.1793
**43**		HAEINEAGR	332–340	0.1789
**44**		SAEAGVDAASVSEEF	345–359	0.1784
**45**		VLLPDEVSGLEQLESIIN	244–261	0.1723
**46**		EVVGSAEAGVDAASVSEEFR	341–360	0.1703
**47**		NVLQPSSVHSQTAM	160–173	0.1699
**48**		AMGITDVFSS	300–309	0.1674
**49**		SSNVMEE	270–276	0.1638
**50**		EVVGSAEAGVDAASVSEEF	341–359	0.1629
**51**		SGISSAESLQ	314–323	0.1544
**52**		ELINSWVESQTNGIIR	144–159	0.1482
**53**		SGISSAESLE	314–323	0.1469
**54**		VTEQESKPVQMM	201–212	0.1453
**55**		AEINEAGR	333–340	0.1437
**56**		VLKPSSVDSQTAM	161–173	0.1431
**57**		VESQTNGIIR	150–159	0.1401
**58**		NVLQPSSVDSQTAM	160–173	0.1362
**59**		VLLPDEVSGLEQLESR	244–259	0.1282
**60**		VLLPDEVSGLEKLESIINFEK	244–264	0.1189
**61**		VASMASEK	220–227	0.1086
**62**		ISQAVH	324–329	0.1070
**63**		TSSNVMEER	269–277	0.1050
**64**		VLLPHEVSGLEQLES	244–258	0.0986
**65**		QITKPNDVY	90–98	0.0968
**66**		DILNQITKPNDVY	86–98	0.0951
**67**		VLLPDEVSGLEQLES	244–258	0.0916
**68**		NQITKPNDVY	89–98	0.0879
**69**		LTEWTSSNVMEER	265–277	0.0807
**70**		VTEQESKPVQMK	201–212	0.0805
**71**		RVTEQESKPVQM	200–211	0.0787
**72**		NQITEPNDVY	89–98	0.0742
**73**		VTEQESKPVQM	201–211	0.0712
**74**		VTEQESKPV	201–209	0.0395
**75**		NVLQPSSVDSQTAMVLVNAIVFK	160–182	0.0365
**76**		NVLQPSSVDSQTAMVLVNAIVF	160–181	0.0218
**77**	Ovotransferrin (45%)	KDSNVNWNNLK	458–468	0.4863
**78**		GEADAVALHGGLVY	406–419	0.4290
**79**		NLQMDDFELL	579–588	0.4262
**80**		NLKMDDFELL	579–588	0.3988
**81**		KDQLTPSPR	352–360	0.3329
**82**		VPSLMHSQLY	329–338	0.3155
**83**		GAIEWEGIESGSVEKAVAK	155–173	0.2986
**84**		KGEADAVALDGGLVY	405–419	0.2793
**85**		TAGVCGLVPVMAER	420–433	0.2662
**86**		VPSLMDSQLY	329–338	0.2612
**87**		AQSDFGVDTK	289–298	0.2525
**88**		GAIEWEGIESGSVEQAVAK	155–173	0.2308
**89**		RVPSLMDSQLY	328–338	0.2249
**90**		VPVMAER	427–433	0.1784
**91**		VAAHAVVAR	266–274	0.1704
**92**		GAIEWEGIESGSVEQAVAE	155–173	0.1683
**93**		AIEWEGIESGSVEQAVAK	156–173	0.1624
**94**		LKPIAAEVY	93–101	0.1209
**95**		KLKPIAAEVY	92–101	0.1184
**96**		HAVVVRPEK	611–619	0.1049
**97**		IQHSTVEENTGGK	559–571	0.1005
**98**		TVNDLQGK	124–131	0.0955
**99**		AVVVRPEK	612–619	0.0953
**100**		TVNENAPDQKDEYELL	231–246	0.0946
**101**		QGIESGSVEQAVAK	160–173	0.0919
**102**		TDERPASY	443–450	0.0891
**103**		IKHSTVEENTGGK	559–571	0.0865
**104**		VAAHAVVARDDNQVEDIW	266–283	0.0847
**105**		DLTQQER	44–50	0.0846
**106**		VQHSTVEENTGGK	559–571	0.0835
**107**		EGIESGSVEQAVAK	160–173	0.0768
**108**		TVISSLK	682–688	0.0710
**109**		HTTVNENAPDQKDEYELL	229–246	0.0630
**110**		EGIESGSVEQAVAE	160–173	0.0600
**111**		VVVRPEK	613–619	0.0580
**112**		TVEENTGGK	563–571	0.0491
**113**	Ovalbumin-related protein Y (32%)	ISDAVHGVF	324–332	0.4692
**114**		MISDAVHGVF	323–332	0.4224
**115**		VLLPDEVSGLEHIEKTINF	244–262	0.2846
**116**		HSLELEEFR	354–362	0.2286
**117**		SLEIADKLY	99–107	0.1898
**118**		VLLPDEVSGLER	244–255	0.1871
**119**		VLLPDEVSGLERIEKTIN	244–261	0.1605
**120**		VLLPDEVSGLERIEK	244–258	0.1316
**121**		MEVNEEGTEATGSTGAIGNIK	333–353	0.1211
**122**		TGGVEEVNFK	127–136	0.1200
**123**		NVATLPAEK	219–227	0.1152
**124**	Ovalbumin-related protein X (38%)	ILELPFASGDLLML *	74–87	0.8643
**125**		TGISSAESLK	158–167	0.1223
**126**		NVATLPAEK	63–71	0.1152
**127**		VLLPDEVSDLER	88–99	0.1084
**128**		ISQAVH	168–173	0.1070
**129**		AGSTGVIEDIK	187–197	0.1025
**130**		VTKQESKPVQM	45–55	0.0833
**131**	Ovomucoid (42%)	FPNATDKEGK	32–41	0.3269
**132**		DLRPICGTDGVTY	49–61	0.2154
**133**		VEQGASVDKR	137–146	0.0903
**134**		VEQGASVDER	137–146	0.0734
**135**	Ovomucin (16%)	DGSGGCIPK *	814–822	0.6103
**136**		VTDSF	1591–1595	0.2004
**137**		SNSLVILTQA	1494–1503	0.1384
**138**		IQEIATDPGAEK	941–952	0.1215
**139**	Cystatin (12%)	LLGAPVPVDENDEGLQR	30–46	0.2450
**(b) Diphenolase inhibitory activity**				
**1**	Ovalbumin (81%)	ADHPFLF *	361–367	0.9699
**2**		MYQIGLFR *	212–219	0.8011
**3**		SMLVLLPDEVSGLEQLESIINFEK *	241–264	0.6983
**4**		FDKLPGFGD *	60–68	0.6817
**5**		HIATNAVLFFGR *	371–382	0.5209
**6**		FKDEDTQAMPFR *	189–200	0.5001
**7**		DEDTKAMPFR	191–200	0.4978
**8**		ILELPFASGTMS	230–241	0.4972
**9**		DEDTQAMPFR	191–200	0.4842
**10**		MLVLLPDEVSGLEQLESIINFEK	242–264	0.4842
**11**		YPILPEYLQCVK	112–123	0.4769
**12**		AFKDEDTQAMPFR	188–200	0.4702
**13**		SSSANLSGISSAESLK	308–323	0.3207
**14**		SQAVHAAHAEINEAGR	325–340	0.2965
**15**		VTEQESKPVQMMYQIGLFR	201–219	0.2886
**16**		GGLEPINFQTAADQAR	128–143	0.2810
**17**		SQTAMVLVNAIVFK	169–182	0.2781
**18**		VASMASEKMK	220–229	0.2728
**19**		AAHAEINEAGR	330–340	0.2679
**20**		YPILPEYLQ	112–120	0.2638
**21**		QAVHAAHAEINEAGR	326–340	0.2606
**22**		ISQAVHAAHAEINEAGR	324–340	0.2599
**23**		EAQCGTSVNVHSSLR	71–85	0.2554
**24**		SANLSGISSAESLK	310–323	0.2537
**25**		SSANLSGISSAESLK	309–323	0.2497
**26**		EVCGSAEAGVDAASVSEEFR	341–360	0.2424
**27**		GLEPINFQTAADQAR	129–143	0.2420
**28**		VLLPDEVSGLEQLESIINFEQ	244–264	0.2410
**29**		VLVNANVFK	174–182	0.2362
**30**		NSQAVHAAHAEINEAGR	324–340	0.2273
**31**		ISQAVHAAHAEIN	324–336	0.2267
**32**		DILNQITKPNDVYSFSLASR	86–105	0.2229
**33**		FQTAADQAR	135–143	0.2202
**34**		VLVNAIVFK	174–182	0.2049
**35**		MAMGITDVFSSSANLSGISSAESLK	299–323	0.1963
**36**		AVHAAHAEINEAGR	327–340	0.1935
**37**		SVNVHSSLR	77–85	0.1930
**38**		LCEWTSSNVMEER	265–277	0.1886
**39**		VLLPDEVSGLEQLESIINFEK	244–264	0.1885
**40**		DLEPINFQTAADQAR	129–143	0.1854
**41**		LSGISSAESLK	313–323	0.1793
**42**		HAEINEAGR	332–340	0.1789
**43**		LEPINFQTAADQAR	130–143	0.1739
**44**		EVVGSAEAGVDAASVSEEFR	341–360	0.1703
**45**		DILNQITKPNDVYSF	86–100	0.1698
**46**		EAGVDAASVSEEFR	437–360	0.1664
**47**		AEAGVDAASVSEEFR	346–360	0.1641
**48**		VHAAHAEINEAGR	328–340	0.1633
**49**		EVVGAAEAGVDAASVSEEFR	341–360	0.1628
**50**		EPINFQTAADQAR	131–143	0.1588
**51**		ISQAVHAAH	324–332	0.1572
**52**		ELINSWVESQTNGIIR	144–159	0.1482
**53**		VTEQESKPVQMM	201–212	0.1453
**54**		VTEQESKPVQMMY	201–213	0.1416
**55**		RVTEQESKPVQMMY	200–213	0.1413
**56**		GITHVFSSSANLSGISSAESLK	302–323	0.1401
**57**		VLVNAIVFE	174–182	0.1401
**58**		AMGNTDVFSSSANLSGISSAESLK	300–323	0.1395
**59**		NVLQPSSVDSQTAM	160–173	0.1362
**60**		EWTSSNVMEER	267–277	0.1280
**61**		GTSVNVHSSLR	75–85	0.1274
**62**		LQPSSVDSQTAMVLVNAIVFK	162–182	0.1259
**63**		AMGITDVFSSSANLSGISSAESLK	300–323	0.1239
**64**		VTEQESKPVKM	201–211	0.1172
**65**		GITDVFSSSANLSGISSAESLK	302–323	0.1089
**66**		VASMASEK	200–227	0.1086
**67**		TSSNVMEER	269–277	0.1050
**68**		DILNQITKPNDVY	86–98	0.0951
**69**		LTEWTSSNVMEER	265–277	0.0807
**70**		ELINSWVESQTN	144–155	0.0798
**71**		TEWTSSNVMEER	266–277	0.0763
**72**		LYAEER	106–111	0.0759
**73**		EAGVDAASVS	347–356	0.0755
**74**		VASMASEE	220–227	0.0721
**75**		VTEQESKPVQM	201–211	0.0712
**76**		TQINK	52–56	0.0607
**77**		NKVVR	55–59	0.0591
**78**		VTEQESKPVQMMYQIGLFRVASMASEK	201–227	0.0512
**79**		NVLKPSSVDSQTAMVLVNAIVFK	160–182	0.0486
**80**		NVLQPSSVDSQTAMVLVNAIVFK	160–182	0.0365
**81**	Ovotransferrin (63%)	SDFHLFGPPGK *	299–309	0.8619
**82**		SGYSGAFHCLK *	208–218	0.8239
**83**		SGAFHCLK *	211–218	0.8038
**84**		CQLCQGSGGIPPEK *	518–531	0.6874
**85**		YFGYTGALRCLV *	540–551	0.6541
**86**		DLLFKDSAIMLK *	216–327	0.6400
**87**		SGALHCLK *	211–218	0.5971
**88**		FMMFESQNKDLLFK *	644–657	0.5892
**89**		YFGYTGALR *	540–548	0.5801
**90**		KDSNVNWNNLK	458–468	0.4863
**91**		DDNKVEDIWSFLSK	275–288	0.4641
**92**		SGGIPPEK	524–531	0.4622
**93**		NIPIGTLLHRG	145–155	0.4328
**94**		DLLFKDSAIMLE	316–327	0.4322
**95**		SAIQSMR	345–351	0.4293
**96**		ANVMDYR	595–601	0.4255
**97**		FFSASCVPGATIEQK	174–188	0.4152
**98**		NIPIGTLLHR	145–154	0.3756
**99**		TSCHTGLGR	132–140	0.3624
**100**		GAIEWEGIESGSVEQAVAKFFSASCVPGA	155–183	0.3503
**101**		FMMFESKNK	644–652	0.3416
**102**		KDQLTPSPR	352–360	0.3329
**103**		FMMFESQNK	644–652	0.3265
**104**		GDVAFVK	222–228	0.3236
**105**		EAGLAPYK	85–92	0.3173
**106**		GLIHNR	488–493	0.3124
**107**		VEDIWSFLSE	278–288	0.3066
**108**		GAIEWEGIESGSVEKAVAK	155–173	0.2986
**109**		DGKGDVAFVK	219–228	0.2939
**110**		SDFGVDTK	291–298	0.2580
**111**		AQSDFGVDTK	289–298	0.2525
**112**		GDVAFIKHSTVEENTGGK	554–571	0.2429
**113**		TDERPASYF	443–451	0.2356
**114**		GAIEWEGIESGSVEQAVAK	155–173	0.2308
**115**		AAHAVVAR	267–274	0.2295
**116**		GANEWEGIESGSVEQAVAK	155–173	0.2076
**117**		GAIEWEGNESGSVEQAVAK	155–173	0.2067
**118**		AQSDFGVDTE	289–298	0.1852
**119**		FYTVISSLKTCNPS	680–693	0.1792
**120**		VAAHAVVAR	266–274	0.1704
**121**		GAIEWEGIESGSVEQAVAE	155–173	0.1683
**122**		FGVHGSEK	634–641	0.1601
**123**		FGVNGSEKSK	634–643	0.1364
**124**		RFGVNGSEK	633–641	0.1351
**125**		GDVAFVQHSTVEENTGGK	554–571	0.1323
**126**		KGTEFTVNDLQGK	119–131	0.1190
**127**		GTEFTVNDLQGK	120–131	0.1166
**128**		DVAFIQHSTVEENTGGK	555–571	0.1158
**129**		KCVAS	531–535	0.1136
**130**		TDERPASY	443–450	0.0891
**131**		LKPIAAEVYEHTEGSTTSYY	93–112	0.0753
**132**		YTVISSLK	681–688	0.0742
**133**		CTVVDETK	390–397	0.0495
**134**		HTTVNENAPDQK	229–240	0.0390
**135**	Ovomucoid (38%)	FPNATDKEGK	32–41	0.3269
**136**		VMVLCNR	107–113	0.2887
**137**		GASVDKR	140–146	0.2203
**138**		SIEFGTNISK	71–80	0.1899
**139**		AVVESNGTLTLSHFGK	194–209	0.1597
**140**		FCNAVVES	191–198	0.1383
**141**		VEQGASVDKR	137–146	0.0903
**142**		CAHKVEQ	133–139	0.0902
**143**		VEQGASVDER	137–146	0.0734
**144**		CNFCNAVVESNGTLTLSHFGK	189–209	0.0623
**145**		VEQGASVDK	137–145	0.0585
**146**		VEQGASVDE	137–145	0.0467
**147**	Ovomucin (26%)	SGGQFSLTSTVKVC *	1973–1986	0.5560
**148**		SSCEDCVCT *	1888–1896	0.5459
**149**		FNCSSAGPGAIGSEC *	771–785	0.5429
**150**		SSCICS *	270–275	0.5397
**151**		FDGRSR *	1000–1005	0.5330
**152**		PAQEQLM	1255–1261	0.3931
**153**		KSLSICSLK	916–924	0.3501
**154**		VTSDGCCK	2001–2008	0.3200
**155**		LEGCYPECS	1163–1171	0.3088
**156**		ECGNSC	312–317	0.2923
**157**		EPSELCK	1629–1635	0.2680
**158**		TCTCNKR	846–852	0.2315
**159**		DTCADPE	319–325	0.2167
**160**		VTDSF	1591–1595	0.2004
**161**		TATGAVEDSAAAFGNSWE	547–564	0.1661
**162**		GTCSTYS	2044–2050	0.1278
**163**		IQEIATDPGAEK	941–952	0.1215
**164**		EVIVDTLLSR	1722–1731	0.1101
**165**		VQVSTVGR	28–35	0.0990
**166**		AVTGTN	1901–1906	0.0685
**167**	Lysozyme (54%)	GYSLGNWVCAAK *	40–51	0.6563
**168**		HGLDNYRG	33–40	0.4763
**169**		HGLDNYR	33–39	0.3753
**170**		GTDVQAWIR	135–143	0.3365
**171**		GILQINSR	72–79	0.2008
**172**		FESNFNTQATNR	52–63	0.1820
**173**		SNFNTQATNR	54–63	0.1487
**174**		ILQINSR	73–79	0.1212
**175**		NTDGSTDYGILQINSR	64–79	0.0900
**176**	Ovalbumin-related protein Y (28%)	VHNLFK	80–85	0.3580
**177**		HSLELEEFR	354–362	0.2286
**178**		VATLPAEKMK	220–229	0.1937
**179**		KFYTGGVEEVNFK	124–136	0.1917
**180**		VLLPDEVSGLER	244–255	0.1871
**181**		FYTGGVEEVNFK	125–136	0.1570
**182**		ATGSTGAI	342–349	0.1262
**183**		TESQMK	50–55	0.0899
**184**	Ovostatin (2%)	EKMAPALRLLV	538–548	0.4863
**185**		LVDKDNSPISNK	379–390	0.1410
**186**	Ovalbumin-related protein X (29%)	HNPTNTIVYFGR	217–228	0.2810
**187**		ILELPFASGDLSMLVLLPDEVSDLER	74–99	0.1682
**188**		ILELPFASGDLSMLVLLPDEVSHLER	74–99	0.1607
**189**		ISQAVHGAFMELSEDGIEMAGSTGVIEDIK	168–197	0.0118

* shortlisted peptides (PeptideRanker score > 0.5) for structure-activity relationship analysis.
